# Case report: Whole-exome sequencing reveals a novel variant in a patient with epilepsy presenting with fever

**DOI:** 10.3389/fgene.2026.1841342

**Published:** 2026-05-21

**Authors:** Wei Guo, Dake Song, Ke Cheng, Chunhui Wang, Yujuan Wang, Xi Yang, Yan Lin, Xun Jiang, Xiyuan Liu, Li Lan

**Affiliations:** 1 Department of Pediatrics, Tangdu Hospital, Fourth Military Medical University, Xi’an, China; 2 Department of Pharmacology, School of Pharmacy, Fourth Military Medical University, Xi’an, China; 3 State Key Laboratory of Military Stomatology, National Clinical Research Center for Oral Diseases, Shaanxi International Joint Research Center for Oral Diseases, Department of Pharmacy, School of Stomatology, Fourth Military Medical University, Xi’an, China; 4 Honghui Hospital, Xi’an Jiaotong University, Xi’an, China

**Keywords:** BK channel, *de novo* variant, epilepsy, KCNMA1, pediatric neurology, whole-exome sequencing

## Abstract

Epilepsy is a prevalent neurological disorder characterized by recurrent seizures and significant impacts on quality of life. This case report describes a 2-year-old male patient presenting with epilepsy associated with fever, in whom whole-exome sequencing (WES) revealed a novel *de novo* variant in the *KCNMA1* gene, resulting in the amino acid substitution p.Leu187His. This variant is classified as likely pathogenic and is situated within a key functional domain of the BK channel, a crucial regulator of neuronal excitability. In silico analyses suggest that the substitution alters the local hydrophobicity and may disrupt the channel’s function, potentially contributing to the patient’s seizure activity. Additionally, the identification of this genetic variant underscores the importance of genetic factors in epilepsy, particularly in cases of drug-resistant epilepsy (DRE) or those with unclear etiology. The findings highlight the utility of WES in diagnosing genetically mediated epilepsy and the need for further research to establish comprehensive genotype-phenotype correlations, ultimately guiding personalized treatment strategies for affected individuals.

## Introduction

Epilepsy is one of the most prevalent chronic neurological disorders worldwide, affecting approximately 50 million individuals across all age groups (World Health Organization, 2022). Characterized by recurrent, unprovoked seizures resulting from abnormal synchronous neuronal activity in the brain, epilepsy imposes substantial burdens on patients’ quality of life, including cognitive impairment, psychological distress, and social disability (Thijs et al., 2019). Epilepsy exhibits extensive etiological heterogeneity, with causal factors varying significantly across age groups—from neonates to adults. Despite advances in antiseizure medications (ASMs), nearly 30% of patients develop DRE, highlighting the need to unravel the underlying etiologies to guide personalized treatment strategies (Mesraoua et al., 2023).As a major acquired cause (notably in low-and middle-income regions), central nervous system (CNS) infections trigger seizures acutely and chronically. Structural brain lesions, metabolic disorders, and cryptogenic epilepsy (unknown cause, 20%–30% of cases) are additional key contributors (Symonds et al., 2021; Manford, 2017).

In recent decades, genetic factors have emerged as a dominant contributor to epilepsy pathogenesis, particularly in pediatric and early-onset cases. Estimates suggest that monogenic variants account for 10%–20% of all epilepsies (Myers, 2022), with next-generation sequencing (NGS) technologies (e.g., WES) enabling the identification of novel disease-associated genes (Chen et al., 2025). Among these, genes encoding ion channels—critical regulators of neuronal excitability—are frequently implicated, as their dysfunction directly disrupts the balance between excitatory and inhibitory signaling in the CNS. The *KCNMA1* gene, located on chromosome 10q22.3, encodes the α-subunit of the large-conductance calcium-activated potassium (BK) channel—also known as KCa1.1. BK channels are widely expressed in the CNS, where they play a pivotal role in modulating neuronal excitability, action potential duration, and neurotransmitter release (Contet et al., 2016; Griguoli et al., 2016). Activated by both membrane depolarization and intracellular calcium (Ca^2+^) elevation, BK channels mediate rapid potassium (K^+^) efflux, which repolarizes the neuronal membrane and limits excessive firing-acting as a “brake” on hyperactivity.

Despite growing recognition of *KCNMA1* as an epilepsy-associated gene, many variants remain reported in small cohorts or single cases, limiting our understanding of genotype-phenotype correlations. Herein, we present a case of epilepsy in which NGS identified a pathogenic variant in *KCNMA1*. This report aims to: (1) expand the clinical and genetic spectrum of *KCNMA1*-related epilepsy; (2) discuss the potential functional consequences of the identified variant in the context of existing literature; and (3) highlight the utility of NGS in diagnosing genetically mediated epilepsy, particularly in patients with DRE or unclear etiologies. Such insights may inform future efforts in genetic counseling, prognostic assessment, and targeted therapeutic development for *KCNMA1*-related disorders.

## Case description

A 2-year-old male patient was admitted to our hospital for treatment due to paroxysmal seizures, which involve loss of consciousness and unresponsiveness. The patient has experienced six consecutive seizures over 4 months. The first five seizures were associated with fever, while the most recent one occurred without any apparent trigger. Each seizure lasts about 10 s before resolving, leaving the child appearing lethargic afterward. Local hospital completed cranial MRI and cerebrospinal fluid examinations, which were normal. For further treatment, the patient was transferred to our hospital.

On routine clinical observation, the patient appeared to be developing normally. Laboratory and auxiliary examinations revealed the following findings: Routine blood test showed thrombocytosis. Plasma ammonia was 132 μmol/L (reference range: 9–30 μmol/L), and plasma lactic acid was 5.29 mmol/L (reference range: 0.5–2.0 mmol/L), indicating significant elevation. Both parents are healthy, with no consanguinity or family history of genetic diseases. A formal developmental assessment using the Erxin Scales II was performed during hospitalization. The patient’s developmental age and developmental quotient (DQ) in each domain were as follows: gross motor 39 months (DQ 83), fine motor 33 months (DQ 71), adaptive behavior 34 months (DQ 74), language 34 months (DQ 74), and personal-social 33 months (DQ 71). The overall developmental age was 35 months, with an overall DQ of 75 (reference: DQ ≥ 85 normal, 70–84 mild to moderate delay, <70 significant delay). These results indicated mild to moderate delays across all domains, with relative preservation of gross motor function and more pronounced difficulties in fine motor, language, and personal-social skills. Video electroencephalogram (EEG) demonstrated abnormalities: 1) The background rhythm was slower with poor continuity compared to age-matched normal children; 2) During both awake and sleep states, generalized high-to-very-high amplitude spike-and-slow waves and slow waves were observed in short bursts.

Clinically, epilepsy or central nervous system infection were initially suspected. However, since the latest episode had no obvious trigger, a hereditary metabolic disease is suspected, and genetic testing is recommended to confirm the diagnosis. This genetic testing was approved by the hospital’s Medical Ethics Committee after obtaining informed consent from the child’s guardian.

Following the identification of the *de novo KCNMA1* p.Leu187His variant by trioWES, several management adjustments were implemented. First, further invasive diagnostic procedures (e.g., repeated lumbar puncture) were deferred, as a genetic etiology had been confirmed. Second, an ASM regimen was initiated with oxcarbazepine, a sodium channel blocker, while avoiding ASMs that may interfere with potassium channel function.

## Methods

Trio whole-exome sequencing (trioWES) was performed on the proband (the patient) and his biological parents to identify potential pathogenic genetic variants. Peripheral venous blood samples were collected from all three subjects, and genomic DNA was extracted using standard commercial kits. The trioWES assay relied on the detection technology provided by Chigene Co., Ltd. (Beijing, China). Protein-coding exome enrichment was conducted using the xGen Exome Research Panel v2.0 (IDT, Iowa, United States). This panel comprises 429,826 individually synthesized and quality-controlled probes, targeting the 39 Mb protein-coding region (19,396 genes) of the human genome and covering 51 Mb of end-to-end tiled probe space. High-throughput sequencing was performed on an MGISEQ-T7 series sequencer, with at least 99% of the target sequence covered. The sequencing was carried out by Chigene (Beijing) Translational Medical Research Center Co., Ltd.

Sequencing data were processed using FastQ to remove adapters and filter low-quality reads. Paired-end reads were then aligned to the GRCh37/hg19 reference genome using the Burrows-Wheeler Aligner (BWA). Base quality scores from HiSeq were recalibrated with the Genome Analysis Toolkit (GATK) Table Recalibration, and variants were called using GATK Unified Genotyper. Variants were annotated with databases for minor allele frequencies (MAFs) (1,000 genomes, dbSNP, ESP, ExAC, and gnomAD). Pathogenicity prediction was conducted using a series of software packages.

Gene structure and protein domain model diagrams were created using Illustrator for Biological Sequences v2.0 (IBS). Protein domain predictions were conducted with InterPro Classification of protein families. Protein morphology and sequence characteristics were visualized using Protter. The hydrogen bond and hydrophobicity of 3D protein images were generated with PyMol 2.5. ProtScale was used to predict the hydrophobicity or hydrophilicity scales.

## Results

### Identification of a de novo *KCNMA1* variant

**FIGURE 1:**
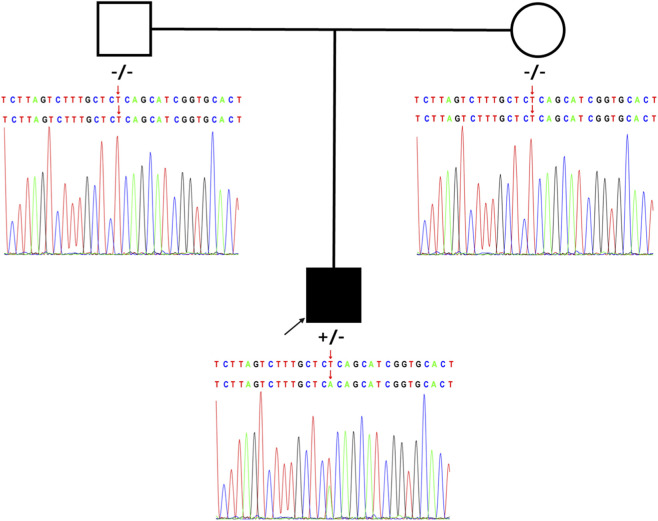
Novel heterozygous *de novo KCNMA1* variant causes epilepsy. Pedigree and Sanger sequencing validation for the *KCNMA1* NM_001161352.2: c.560 (exon3)T>A (p.Leu187His) variant in an affected individual compared with healthy parents.

Trio-based whole-exome sequencing (trio-WES) identified a heterozygous missense variant in the proband, c.560T>A in exon 3 of the *KCNMA1* gene (NM_001161352.2), resulting in the amino acid substitution p.Leu187His. Segregation analysis confirmed that this variant occurred *de novo*, as both parents were wild-type for this locus (Figure 1). According to the American College of Medical Genetics and Genomics (ACMG) guidelines, this variant was classified as Likely Pathogenic based on the following evidence codes: PS2 (*De novo* in a patient with the disease and no family history), PM1 (Located in a mutational hot spot and/or critical and well-established functional domain), PM2_Supporting (Absent from population databases), PP2 (Missense variant in a gene that has a low rate of benign missense variation), and PP3 (Multiple lines of computational evidence support a deleterious effect). The computational scores supporting PP3 are as follows: SIFT score 0.00 (deleterious), PolyPhen-2 score 0.993 (probably damaging), CADD Phred score 28.5 (among the top 1% most deleterious variants in the genome), REVEL score 0.942 (pathogenic, threshold >0.5), and MutationTaster2021 probability 1.000 (disease-causing).

### Gene structure and evolutionary conservation

**FIGURE 2:**
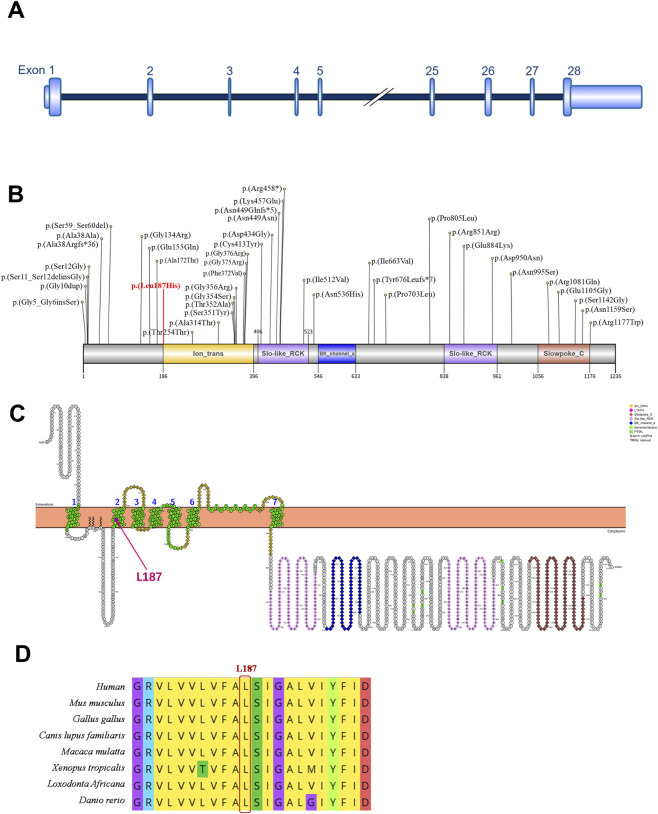
Pattern diagram of the *KCNMA1* gene structure, protein domain, transmembrane domain and protein sequence conserved analysis. **(A)** *KCNMA1* gene (NM_001161352.2) contains 28 exons, and exons 2–28 are involved in gene coding. **(B)** Pattern diagram of the four protein domains: Ion_trans (orange), Slo-like_RCK (purple), BK_channel_a (blue) and Slowpoke_C (pink) and the currently reported missense variant sites that cause epilepsy and the sites in this study (red font). **(C)** Membrane topology plot of KCNMA1 by PROTTER showing Slo-like_RCK (purple), BK_channel_a (blue) and Slowpoke_C (pink) are located inside the cell membrane, Ion_trans (orange) is a transmembrane structure. L187H variant site (red arrow) is in the second transmembrane region. **(D)** Multiple sequence alignment of KCNMA1 proteins from 8 species shows that the L187H variant site is within the conserved sequence.

The *KCNMA1* gene is located on chromosome 10 and comprises 28 exons, with a portion of exon 1 and the entirety of exons 2–27 constituting the coding sequence (Figure 2A). This gene encodes the α-subunit of the large-conductance calcium-activated potassium (BK) channel. The α-subunit mainly has the following four domains: Ion_trans (orange), Slo-like_RCK (purple), BK_channel_a (blue) and Slowpoke_C (pink). Figure 2B shows the reported missense variants in this gene. The resulting protein contains 1236 amino acids and includes key functional domains: an ion transport domain (Ion_trans) with six transmembrane segments (S1-S6), and a large intracellular C-terminal structure composed of the BK_channel_a, Slowpoke_C, and Slo-like_RCK domains (Figure 2C). The p.Leu187His variant is situated within the second transmembrane segment (S2) of the Ion_trans domain (indicated by a red arrow in Figure 2C). Multiple sequence alignment across eight species (Human, Mouse, Chicken, Dog, Macaque, *Xenopus* tropicalis, African elephant, and Zebrafish) using Mega11 demonstrated that the Leu187 residue is highly evolutionarily conserved (Figure 2D), underscoring its functional importance.

### In silico analysis of structural and functional consequences

**FIGURE 3:**
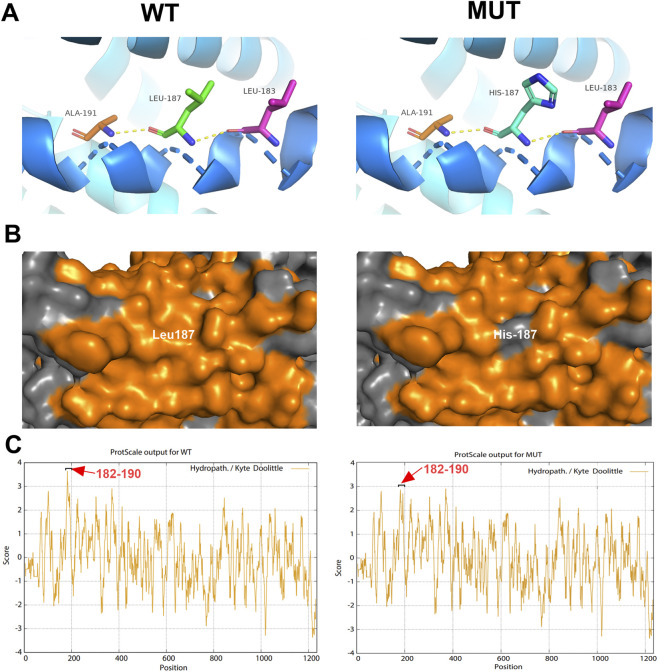
The effect of the variant on the *KCNMA1*-encoded protein function. **(A)** Virtual three-dimensional structure models of the variant analysis were performed with Pymol. The native Leu187 and mutated His187 residues are highlighted in green or blue stick format. Dotted blue lines represent hydrogen bonds. **(B)** The hydrophobic surface distribution map of the protein shows that the hydrophobic amino acid Leu187 is replaced by the hydrophilic amino acid His187. **(C)** The ProtScale website predicts the change in hydrophobicity of the protein before and after variant. The hydrophobicity of the amino acid residues in the 182–190 segment is significantly reduced.

To further investigate the potential impact of the p.Leu187His variant, we performed *in silico* analyses of the protein’s structure and physicochemical properties. The wild-type (WT) and mutant protein structures were modeled using SWISS-MODEL and visualized with PyMOL. Analysis of hydrogen bonding revealed that the Leu187 residue naturally forms two hydrogen bonds with the side chains of Ala191 and Leu183. Notably, the p.Leu187His variant did not alter the overall hydrogen bond network at this position (Figure 3A). However, a significant change was observed in the local hydrophobicity profile. Leu is a strongly hydrophobic amino acid, whereas His is hydrophilic. The p.Leu187His substitution not only altered the hydrophobicity at residue 187 but also perturbed the hydrophobicity pattern across the entire 182–190 amino acid residue segment of the protein side chain (Figures 3B,C). This change is predicted to disrupt the local protein environment, potentially leading to abnormal spatial conformation, reduced stability, and ultimately, impaired functional activity of the BK channel.

In summary, the identified *de novo* p.Leu187His variant is a likely pathogenic variant that interferes with the protein’s side-chain architecture, which may consequently alter the function of the *KCNMA1*-encoded BK channel.

## Discussion

This report describes a 2-year-old male with fever-associated epilepsy and neurodevelopmental delays, in whom trio whole-exome sequencing identified a *de novo* heterozygous missense variant in *KCNMA1* (c.560T>A, p.Leu187His). Classified as likely pathogenic per ACMG guidelines, this variant expands the genetic spectrum of *KCNMA1*-related epilepsy and offers critical insights into genotype-phenotype correlations, the functional role of BK channels in seizure pathogenesis, and the utility of NGS in undiagnosed epilepsies.

The *KCNMA1*-encoded BK channel α-subunit is essential for regulating neuronal excitability: its activation mediates K^+^ efflux, repolarizing membranes and constraining excessive firing—an effect particularly critical in seizure-prone brain regions (hippocampus, cortex, thalamus) (Tabarki et al., 2016). The p.Leu187His variant localizes to the second transmembrane segment of the Ion_trans domain, a region integral to channel folding, membrane anchoring, and ion permeation (Meredith, 2024). While *in silico* analysis showed preserved hydrogen bonding between the mutant residue and adjacent Ala191/Leu183, the substitution of hydrophobic Leu with hydrophilic His disrupts the local and regional (residues 182–190) hydrophobic profile of the transmembrane domain. This perturbation is biologically relevant: transmembrane segments rely on hydrophobic interactions to maintain stable integration into lipid bilayers (De Marothy and Elofsson, 2015; Janoschke et al., 2021). A hydrophilic residue at position 187 may compromise channel trafficking to the plasma membrane, alter pore conformation, or impair voltage/Ca^2+^ sensing—all of which would reduce BK channel activity and disrupt the excitatory-inhibitory balance in the CNS, a hallmark of epilepsy pathogenesis.

Notably, evolutionary conservation of Leu187 across 8 species (including non-mammalian taxa like *Xenopus* tropicalis and *Danio rerio*) further supports its functional significance. Conserved residues in transmembrane domains are rarely tolerant of missense changes, as evidenced by prior studies linking *KCNMA1* missense variants in S6 segments to BK channel dysfunction and early-onset epilepsy (Geng et al., 2023; Liang et al., 2019). While direct electrophysiological validation (e.g., patch-clamp studies of mutant BK channels in HEK293 cells) was not performed here, the combination of ACMG criteria, structural modeling, and conservation data strongly suggests the variant is pathogenic.

The patient’s clinical features—fever-triggered seizures, subsequent unprovoked seizures, and neurodevelopmental delays (language, gross motor, social domains)—align with and extend the phenotypic spectrum of *KCNMA1*-related epilepsy. Previous case series have reported *KCNMA1* variants in patients with developmental and epileptic encephalopathy (DEE), focal seizures, and drug resistance. Fever has been documented as a potential seizure trigger in some instances. For example, Al-Attas et al. described a patient with *KCNMA1*-related refractory status epilepticus in whom fever appeared to exacerbate seizure frequency and severity (AL-attas et al., 2022). Fever may exacerbate the functional deficit of the mutant BK channel: elevated temperatures alter membrane fluidity and ion channel gating kinetic, and a destabilized transmembrane domain (as predicted here) could render the mutant channel more susceptible to thermal stress—triggering seizure activity. This mechanism may explain why the patient’s first five seizures were fever-associated, with later unprovoked seizures reflecting progressive neuronal hyperexcitability as the CNS matures. Of note, Yao et al. identified a transmembrane domain *KCNMA1* variant (E155Q) in a febrile seizure cohort, which supports the potential link between BK channel dysfunction and fever-associated seizures (Yao et al., 2021). Nevertheless, direct evidence linking transmembrane domain variants to temperature sensitivity remains limited, and the proposed mechanism requires further experimental validation.

Although the patient’s normal cranial MRI and cerebrospinal fluid (CSF) findings rule out structural or infectious etiologies, elevated plasma ammonia and lactate may be transient consequences of seizure activity and febrile stress rather than indicators of a primary metabolic or mitochondrial disorder. Although BK channels have been implicated in mitochondrial function in animal models, the present case does not provide direct evidence for such a link (Gao et al., 2023).

Determining whether a *KCNMA1* variant leads to loss-of-function (LoF) or gain-of-function (GoF) is critical for understanding pathogenesis and guiding treatment. Only two GoF variants have been electrophysiologically confirmed (D434G and N999S), both presenting with paroxysmal dyskinesia as a core feature (Bailey et al., 2019). In contrast, most epilepsy-associated *KCNMA1* variants are LoF and localize to transmembrane segments (S1–S6) (Park et al., 2022; Chen et al., 2025). The p.Leu187His variant resides in the S2 transmembrane segment and is predicted to be LoF because: (1) the hydrophobic-to-hydrophilic substitution disrupts local hydrophobicity, likely impairing channel trafficking or voltage sensing; (2) a large Chinese cohort showed that transmembrane variants are strongly associated with isolated epilepsy (66.7%) rather than dyskinesia (Meredith, 2024), consistent with our patient’s phenotype. Therefore, the p.Leu187His variant most likely exerts its pathogenic effect via an LoF mechanism, though direct patch-clamp functional studies are required for definitive confirmation.

The patient is currently only 2 years old, and his current phenotype of fever-associated seizures likely represents an early stage of an evolving neurological syndrome. *KCNMA1* variants are known to be associated with age-dependent phenotypic expansion, including DEE, paroxysmal movement disorders (e.g., chorea, dyskinesia), and progressive cognitive decline, which may manifest later in childhood. Therefore, long-term longitudinal follow-up is essential to fully delineate the clinical spectrum and developmental trajectory associated with the p.Leu187His variant.

This case underscores the value of trioWES in resolving cryptogenic epilepsy, particularly in pediatric patients with atypical features and negative initial workup. Prior to genetic testing, the patient was suspected of having CNS infection or metabolic disease—common differential diagnoses in febrile seizures—but NGS identified a monogenic cause that would have otherwise been missed. For clinical practice, this has three key implications: (1) early NGS testing can reduce unnecessary investigations (e.g., repeated CSF analyses) and shorten diagnostic odysseys; (2) confirmation of a *de novo KCNMA1* variant allows for accurate genetic counseling (low recurrence risk for parents, ∼1% gonadal mosaicism risk; and (3) it guides personalized management—for example, avoiding antiseizure medications (ASMs) that may further impair BK channel function and prioritizing ASMs that enhance inhibitory signalin.

## Data Availability

The datasets presented in this article are not readily available because of legal restrictions under the Chinese Human Genetic Resources Administration Regulations, which prohibit the direct transfer of raw human sequencing data to public repositories without government approval, and because the participant’s informed consent did not authorize public data sharing. Requests to access the de-identified whole-exome sequencing data (including VCF files and genetic testing reports) should be directed to the corresponding author at dk840385597@163.com or 418893819@qq.com.
